# A Window into the Nubian Diet: A Case Study of Food Crop Storage in the Kingdom of Dongola (Fourteenth to Eighteenth Centuries CE), Northern Sudan

**DOI:** 10.1007/s10437-025-09622-y

**Published:** 2025-04-26

**Authors:** Mohammed Nasreldein, Simone Riehl, Agata Deptuła, Lorenzo de Lellis, Artur Obłuski

**Affiliations:** 1 Institute for Archaeological Sciences, University of Tübingen, Hölderlinstrasse 12 72074, Tübingen, Germany; 2 Department of Archaeology, Faculty of Arts and Human Sciences, University of Gezira, Wad Madani, Sudan; 3 Senckenberg Centre for Human Evolution and Paleoenvironment (SHEP), Hölderlinstrasse, 12 72074, Tübingen, Germany; 4 Polish Centre of Mediterranean Archaeology, University of Warsaw, 69 Prosta St., 00‑838 , Warsaw, Poland

**Keywords:** Nubian cuisine, Storage, Sorghum, Porridge, Flatbread, Funj

## Abstract

This paper presents a unique archaeobotanical discovery of stored crops from two domestic structures at Old Dongola in Northern Sudan, dating to the fifteenth to sixteenth centuries CE. The findings provide new insights into aspects of cuisine during the early Funj period (1504–1821 CE) and its historical roots. The assemblage of stored crops—including sorghum, bread wheat, hulled barley, grass pea, cowpea, and radish seeds—reveals a blend of Mediterranean and African influences, reflecting the diverse cultural spheres that converged at Old Dongola. We argue that these crops constituted a fundamental component of the local diet and served as the primary carbohydrate sources for the inhabitants. The size of the storage vessels and containers suggests small-scale storage practices aimed at daily subsistence. Moreover, the location of these stored crops within domestic spaces indicates a household-based economy, in which crop processing and storage were organized at the individual household level.

## Résumé

Cet article présente une découverte archéobotanique unique de récoltes entreposées dans deux structures domestiques à Old Dongola, dans le nord du Soudan, datant des 15e- 16e siècles de notre ère. Les découvertes permettent de mieux comprendre certains aspects de la cuisine au début de la période Funj (1504–1821 de notre ère) et ses racines historiques. L'assemblage des récoltes entreposées—dont le sorgho, le blé panifiable, l’Orge vêtue, gesse commune, dolique à vache, et les graines de radis—révèle un mélange d'influences méditerranéennes et africaines, reflétant les diverses sphères culturelles qui convergeaient à Old Dongola. Nous proposons que ces récoltes constituaient un élément fondamental du régime alimentaire local et servaient de sources principales d'hydrates de carbone pour les habitants. En outre, l’emplacement de récoltes entreposées et leurs récipients suggère une économie fondée sur les ménages dans laquelle la préparation et le conservation des récoltes étaient organisés au niveau de chaque ménage.

## Introduction

Studies on ancient diet are essential for reconstructing the history of important but often overlooked civilizations in the Middle Nile Valley, offering insights into sustainability, adaptation, and cultural exchange across time. The overall image of food studies in Sudan, as previously argued by Ryan et al. (2022), indicates that Sudan has a notable scarcity of ethnographic studies delving into traditional agricultural systems and food practices, except for a few studies concentrated mainly on specific regions, technologies, or cereals (e.g., Al-Batal, 1994; Bacon, 1949: 302–400; Dirar, 1993; Edwards, 2003; Fuller & Carretero, 2018: 109–121; Haaland, 2007: 165–182; Lancelotti et al., 2019; Matthews & Nowotnick, 2019: 465–486). In recent years, there has been a growing interest in archaeobotanical research in Sudan, with several studies emerging from different archaeological sites focusing on plant subsistence and the agricultural economy (Beldados, 2015; Clapham, 2019: 83–101; Fuller, 2015: 33–59; Fuller & Edwards, 2001; Fuller & Lucas, 2020: 928–953; Nasreldein et al., 2024; Out et al., 2016).

**Fig. 1:**
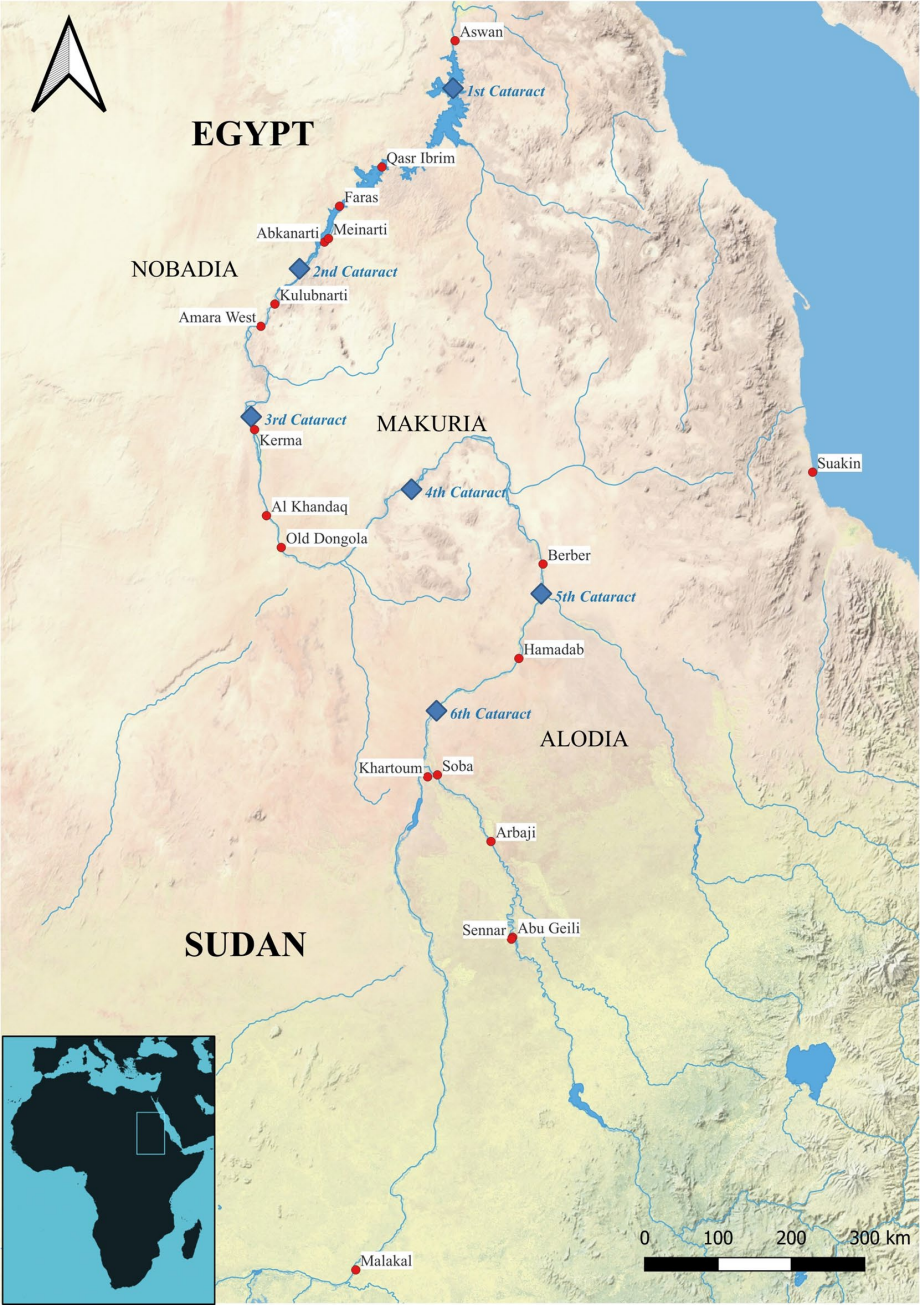
Location of Old Dongola and the medieval Nubian Kingdoms, including major archaeological sites along the Sudanese Nile Valley (prepared by Adrian Chlebowski)

While these contributions have advanced our understanding of Sudanese subsistence strategies, it is important to note that many of these studies remain limited in scope, and substantial gaps persist. Additionally, several recent archaeobotanical investigations at key Sudanese sites, including Jebel Barkal, Old Dongola, Soba, Hamadab, Shaqadud, and Jebel Moya, are still awaiting final publication (Fig. 1). As a result, our knowledge of subsistence practices during key historical periods, particularly the Makurian (fifth to thirteenth centuries CE) and Funj (sixteenth to eighteenth centuries CE) periods, remains incomplete. The only exception is the extensive recognition and cataloguing of sorghum-based beverages, flatbreads, and porridges in Sudan (Anderson & Ahmed, 2006; Bacon, 1949: 302–400; Dirar, 1993; Edwards, 2003: 145–147; Haaland, 2007: 177; Anderson et al., 2007; Matthews & Nowotnick, 2019; Pope, 2013; Ryan, 2018; Ryan et al., 2022), with documentation of other food items and crops remaining limited.

Studies related to food storage practices usually indicate ancient societies’ economic, social, and political conditions. The reasons, nature, and motives for storage among ancient cultures have been a topic of interest among archaeologists for decades (Brenton, 1988; Castellano, 2018; Christakis, 1999; Hildebrand & Schilling, 2016; Kent, 1999; Smyth, 1989; Wesson, 1999). The management and redistribution of stored surplus food is influenced by specific social relations, which shape storage strategies (Psaraki et al., 2013). One of the significant issues in understanding ancient food storage is its visibility in the archaeological record (Groenewoudt, 2009; Kent, 1999). Archaeologists studying food storage in Sudan face limited available data due to an absence of direct evidence of stored crops (Fuller & Edwards, 2001; Rowley-Conwy, 1989: 131–138), with only a few studies discussing storage practices during different historical periods (e.g., Abdalla, 2023: 96–77; Choimet, 2023: 401–421; Tahir et al., 2015: 84–92).

The oldest evidence of physical storage in Sudan was discovered in Khartoum Mesolithic sites dating to c. 9000–6500 BC, represented by large vessels (Haaland, 1995: 161; Khabir, 1987: 377; Magid & Caneva, 1998). Further evidence of crop storage in jars (locally known as *gossi* or *gossiba*) has been identified at multiple sites across Northern Sudan, dating back to around 6000 BC (Bonnet, 1992; Garcea & Hildebrand, 2009; Garcea et al., 2016; Hildebrand & Schilling, 2016; Honegger, 2003, 2004; Spencer, 1997; Welsby, 2008; Wolf et al., 2014). This type of storage persisted in Nubia until the 1990 s, when modernization led to the cessation of *gossi* construction and the decline of traditional grain storage practices (Tahir et al., 2015). Table 1 presents a chronological overview of Sudanese storage practices based on previous studies (summarized in Table 1), illustrating how storage methods evolved from early jar storage to larger-scale granaries and silos. While these studies provide important insights into storage traditions, they often lack detailed explanations regarding key parameters such as the number and size of storage containers in relation to settlement size, the spatial distribution of storage spaces (e.g., household or communal storage), and the taxonomic identification of stored crops. These gaps highlight the need for further investigation into the organization of subsistence strategies and agricultural practices during the Makurian (fifth to thirteenth centuries CE) and Funj (sixteenth to eighteenth centuries CE) periods.

**Table 1:** Overview of the history of food storage practices in Sudan (After, Hildebrand & Schilling, 2016; Tahir et al., 2015; and own research)

Site and date	Type of storage	Archaeological evidence	References
Wadi Kubbaniya 18,000 BC	Delayed consumption	Harvesting *Cyperus* tubers triggered denser regrowth. The tubers required grinding or pounding. Surplus fish and tubers were possibly roasted or dried for later consumption	(Gautier & Van Neer, 1989; Hillman, 1989; Hillman et al., 1989; Wendorf & Schild, 1998)
Khartoum Mesolithic sites 9000–6500 BC	Possible small-scale physical storage	Large ceramic vessels at Khartoum Mesolithic sites along the Nile may have been storage containers. However, there is no evidence for large-scale physical storage or environmental storage	(Haaland, 1995; Khabir, 1987; Magid & Caneva, 1998)
Sai 8-B- 10 C 6000 BC	Possible small-scale physical storage	Pits were found in a large excavation area along with hearths and post-holes aligned in hut configurations	(Garcea, 2006; Garcea & Hildebrand, 2009; Hildebrand & Schilling, 2016)
Khartoum Neolithic sites 5800–5000 BC	Environmental storage. Defined by Hildebrand and Schilling (2016: 92) as “herding of cattle and tethering of barbary sheep in a variable environment”	Grinding of wild cereals is prominent at some base campsites. Seasonal fishing and herding activities dominate at the Zakiab site	(Caneva & Santucci, 2006; Haaland, 1987, 1989; Krzyżaniak, 1991; Mohammed-Ali, 1982)
El Baraga Mesolithic Site 6000–10,000 BC	Storage pits	Surplus food production indicated by storage pits near Kerma	(Honegger, 2004)
Tkulainos Neolithic Site 6000–4000 BC	Storage pits	In eastern Sudan, the Neolithic period was marked by settlement, farming-based food production, and social organization—storage pits	(Sadr, 1991)
Pre-Kerma period 3500–2500 BC	Storage pits	Organized society with increased food production. Large numbers (300) of storage pits in Kerma’s eastern cemetery and circular pits in Kerma city	(Bonnet, 1992; Honegger, 2003)
Sai Island 2700 BC	Ceramic pots in pits	Ceramic pots dating back to 2700 BC were found in deep pits. Some pits contained wheat and barley grains, with possible wooden plank covers	(Geus, 1998)
C-Group sites 2200–1200 BC	Grain storage pits	Increased reliance on agriculture and animal husbandry. Grain storage pits were discovered in lower Nubia with tethering posts and silos inside houses	(Adams, 1977; Emery & Kirwan, 1935)
Kerma period 2500–1500 BC	Brick-supported grain storage pits	Stable communities with well-known grain storage. Grain storage pits were discovered on the bank of Wadi Khewi in Gism Arba’a village and near Kerma cemetery on Sai Island, which had brick-supporting walls	(Gratien, 2006; Osman, 2014)
New Egyptian Kingdom Settlement 1650–1000 BC	Pottery jars and storage pits	Large pottery jars and storage pits were found inside a room in Amara West town	(Spencer, 1997; Spencer et al., 2014)
Kushite Period 650 BC to 350 CE	Silos, large storage mud-jars	Large-scale grain storage was observed at Napatan and Meroitic sites. Discoveries include silos, large jars, and storage mud jars in El Kawa town, Gala Abu Ahmed, Sanam Abu Dom, and other locations	(Choimet, 2023; Jesse, 2010; Onderka et al., 2013, 2018; Vincentelli, 2014; Welsby, 2008; Wolf et al., 2014)
Post-Meroitic Period 350–600 CE	Large storage mud-jars	Abdul Majeed (2012) reported large jars used for grain storage in the El Haraz region (Fourth Cataract)	(Abdul Majeed, 2012)
Christian and early Islamic Periods 600–1821 CE	Various storage facilities	Grain storage practices continued through the medieval Christian and Islamic periods. Discoveries include grain storerooms, storage pits, and mud-built containers known as *gossi* in locations such as Soba, Old Dongola, Kulubnarti’s Christian complex, New Amri, Simit Island, El Fasher market in El-Khandaq, and El Hamra the Christian complex kitchen in the El Ga’ab Depression. Grain storage practices were common in Western Sudan in the Nuba mountains at Jebel Damik, known as El-Siwiba	(Abdalla, 2023; Abdul Majeed, 2012; Adams, 2011; Godlewski, 2013, 2018; Obłuski & Dzierzbicka, 2022; Osman & Edwards, 2012; Shinnie, 1955; Soghayroun, 2014; Tahir et al., 2015; Welsby, 1991)

At Old Dongola, the remains of food storage containers are very common and are key features in almost every household (Obłuski & Dzierzbicka, 2021: 240; Wyżgoł & Deptuła, 2023). Excavations revealed small, medium, and large sized jars (*gossi* in the Nubian language) (Danys, 2022: 90) or baskets (Warowna, 2022: 203) that were used as storage containers for food crops; some of them still in situ. Nevertheless, Old Dongola has yet to undergo a comprehensive study to assess the quantity of stored crops and explore their implications and economic significance for the inhabitants, an exception being the study by Wyżgoł (2022: 112–113), who attempted to estimate the number of people that could have been fed using the grain storage in some houses from the Funj period at Old Dongola.

This paper aims to shed light on the domestic practices of food crop storage during a limited period of time within the Kingdom of Dongola and the Funj periods (fourteenth to eighteenth centuries CE) at Old Dongola. It focuses on small-scale storage assemblages for daily food consumption from two houses at Old Dongola, dated to the fifteenth and sixteenth centuries CE. In particular, these two houses provided a unique example of stored crops intended for daily consumption. We aimed to align and compare our findings with the Sudanese historical and ethnographic data. While some of these crops, such as sorghum, are well-documented, others are only documented on a smaller scale, such as wheat and barley, while the remainder are absent from the historical record of Sudanese cuisine. Our study will provide valuable insights into continuity and change in food crop consumption over time. We also aim to explore the cultural influences represented by different crop types. Our research contributes to filling a research gap by providing new insights into subsistence strategies during the Funj period, an area that remains underexplored.

## The Archaeological Site of *Tungul* (*Dunqula*, Old Dongola)

The archaeological site of Old Dongola (Old Nubian *Tungul*, Arabic *Dunqula*—*دنقلا*) is located in Sudan’s Northern State province (N 18.1323°, E 30.4438°) (Fig. 1). It is situated on the eastern riverbank at the southern outskirt of the Letti Basin, formed by a Nile paleochannel, offering vast agricultural potential. The location of Old Dongola at the end of the Wadi Hawar, an important sub-Saharan communication route, facilitated its growth (Obłuski et al., 2022). According to Godlewski (2013), the city was founded in the fifth to early sixth centuries by one of the first kings of Makuria.

Old Dongola became the capital of Makuria (500–1300 CE). As the city developed, its size reached approximately 200 ha in its heyday between the tenth and twelfth centuries (Obłuski et al., 2022: 260). At the end of the thirteenth century, the regional political situation deteriorated after the Mamluk takeover of Egypt (Obłuski et al., 2022). Following the Mamluk invasion in 1276, the Makurian state was subdued by Egypt and became a vassal state. Makurian kings relinquished parts of the territory to the sultan, paid the annual tribute, and later imposed a *jizya* tax on Christians (a tax historically levied on non-Muslim subjects under Islamic rule as a form of tribute or protection tax). Historical sources refer to the kings of Makuria as governors, while the name Makuria disappears from Arabic sources, and the Kingdom of Dongola is used instead. Makurian elites tried to fight off Mamluk control for almost the entire coming century. Mamluk interest in Makuria gradually declined, and around the mid-fourteenth century, the situation became more complex with the migration of various tribes into the Middle Nile Valley (Obłuski, forthcoming). During the Funj Period (1504–1821 CE), the kingdom of Dongola was an essential part of the Funj Sultanate, often involved in power struggles. The kingdom’s end is linked to the *Shaiqiya* invasions in the eighteenth century. In the 1780s, the last king of Dongola moved out of the city and ceased to use the royal title (Obłuski, forthcoming).

Archaeological work in Old Dongola began in 1964 by the Polish Centre of Mediterranean Archaeology of the University of Warsaw (Godlewski, 2013). In 2018, a new phase of archaeological investigations began in Old Dongola, headed by Artur Obłuski and funded by the European Research Council, abbreviated as the ERC-UMMA Starting Grant project. One of the main objectives of the UMMA project is to investigate the impact of migration on the social structure of the inhabitants after the collapse of the Makurian kingdom (see Obłuski et al., 2022; Obłuski & Dzierzbicka, 2021, 2022).

## Materials and Methods

**Fig. 2:**
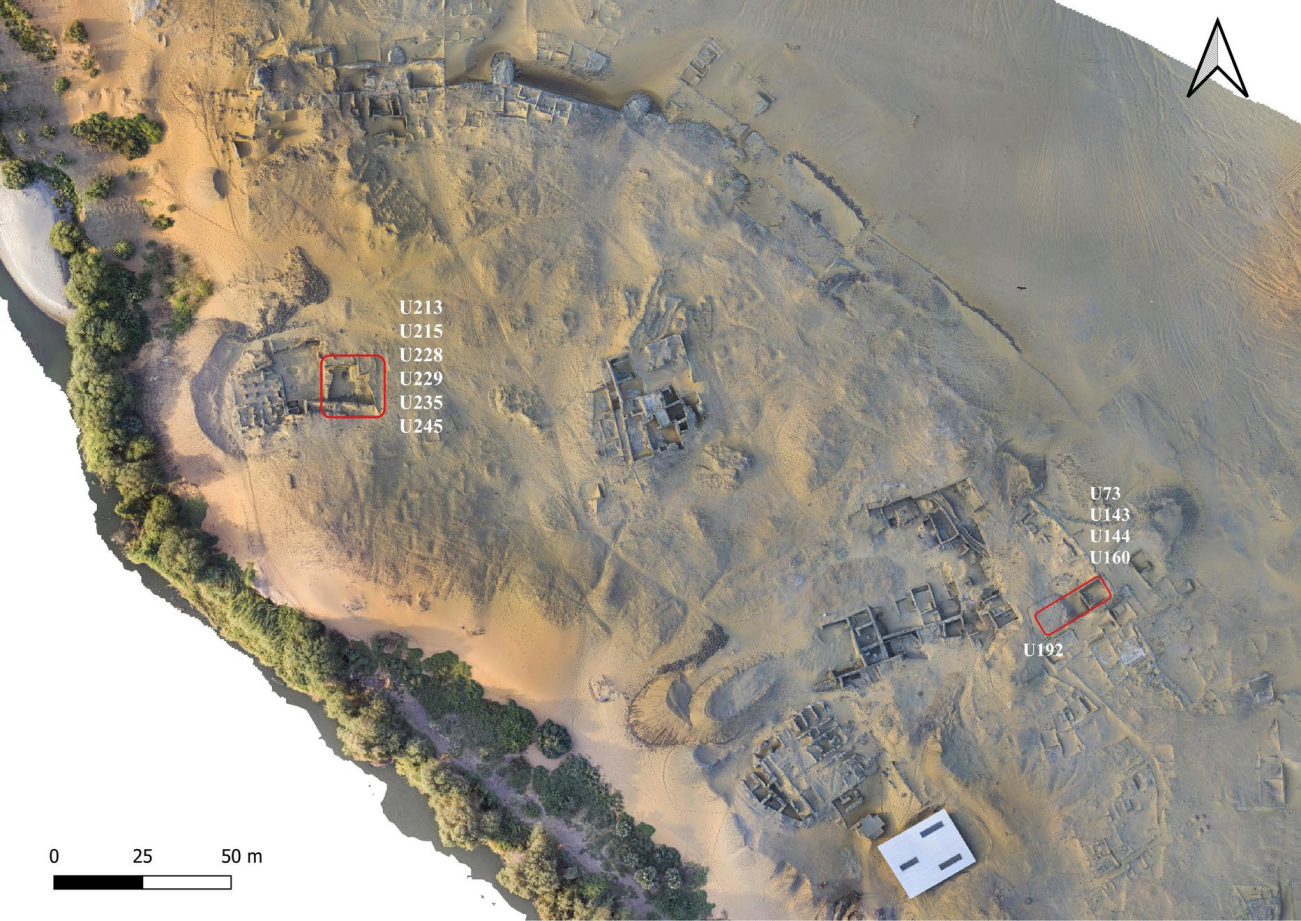
Orthophoto map of the citadel of Old Dongola, highlighting the locations of the two houses discussed in this paper (prepared by Adrian Chlebowski)

**Fig. 3:**
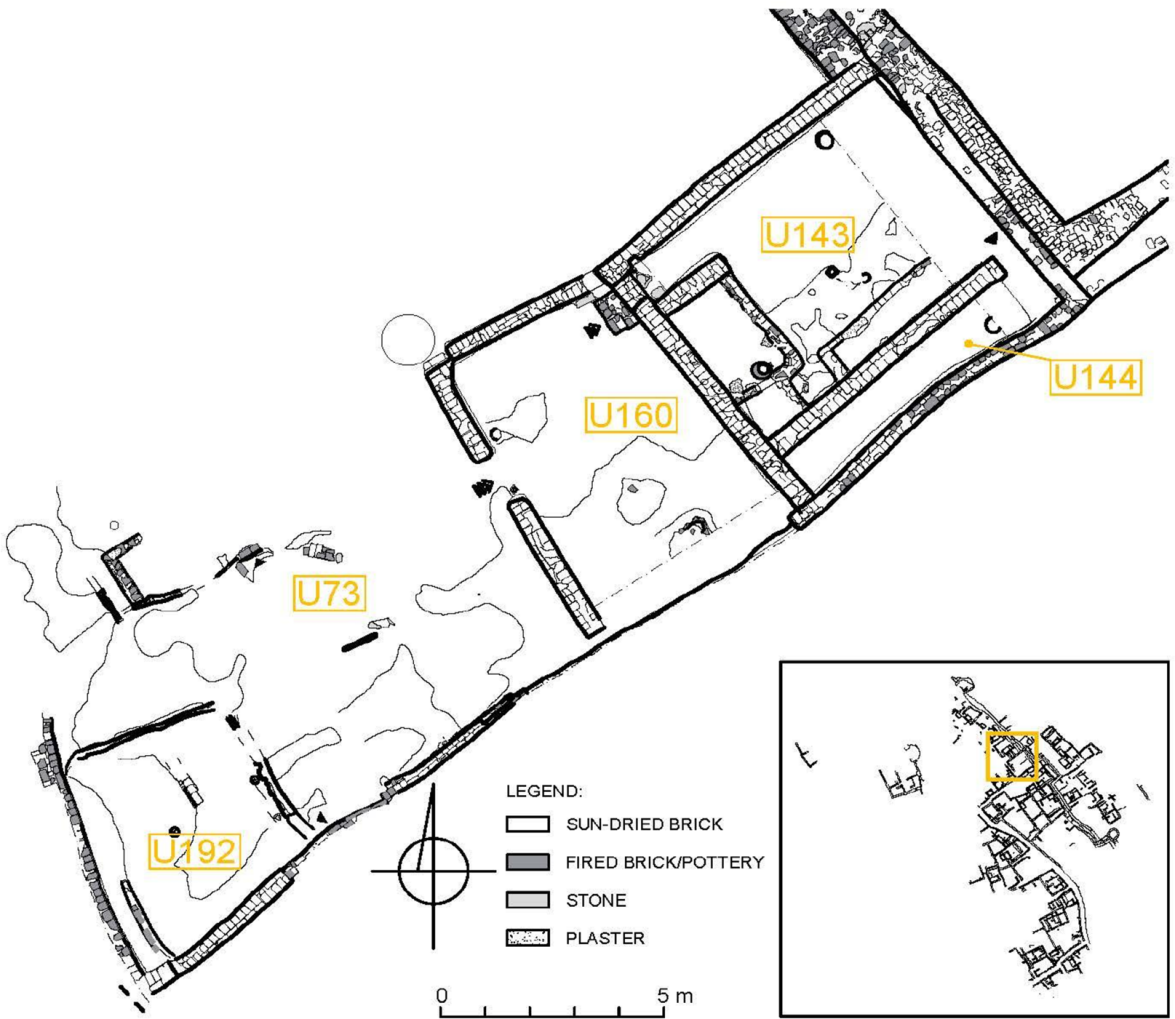
Compound U73/143/144/160/192 (drawing: A. Wujec, A. Chlebowski)

**Fig. 4:**
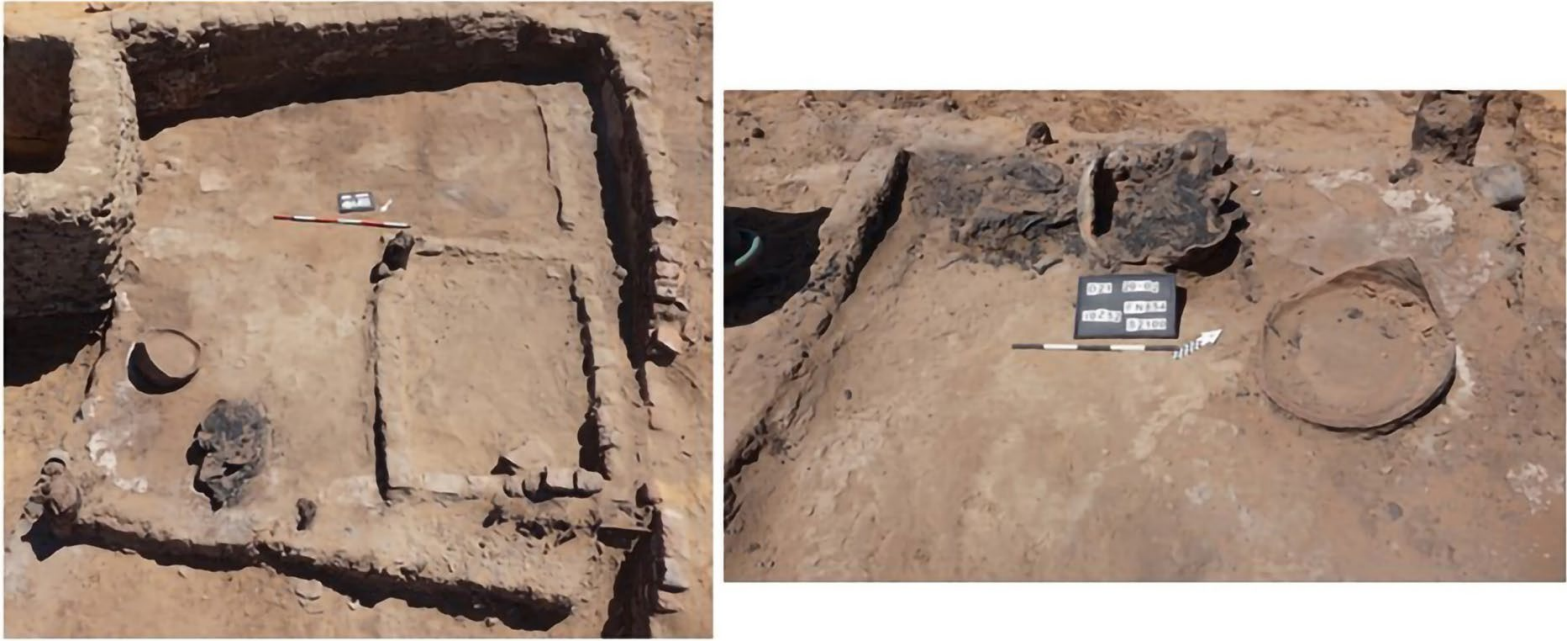
General views of U192, showing the burnt seeds in situ from contexts 1195 and 1199 (photo A. Deptuła)

**Fig. 5:**
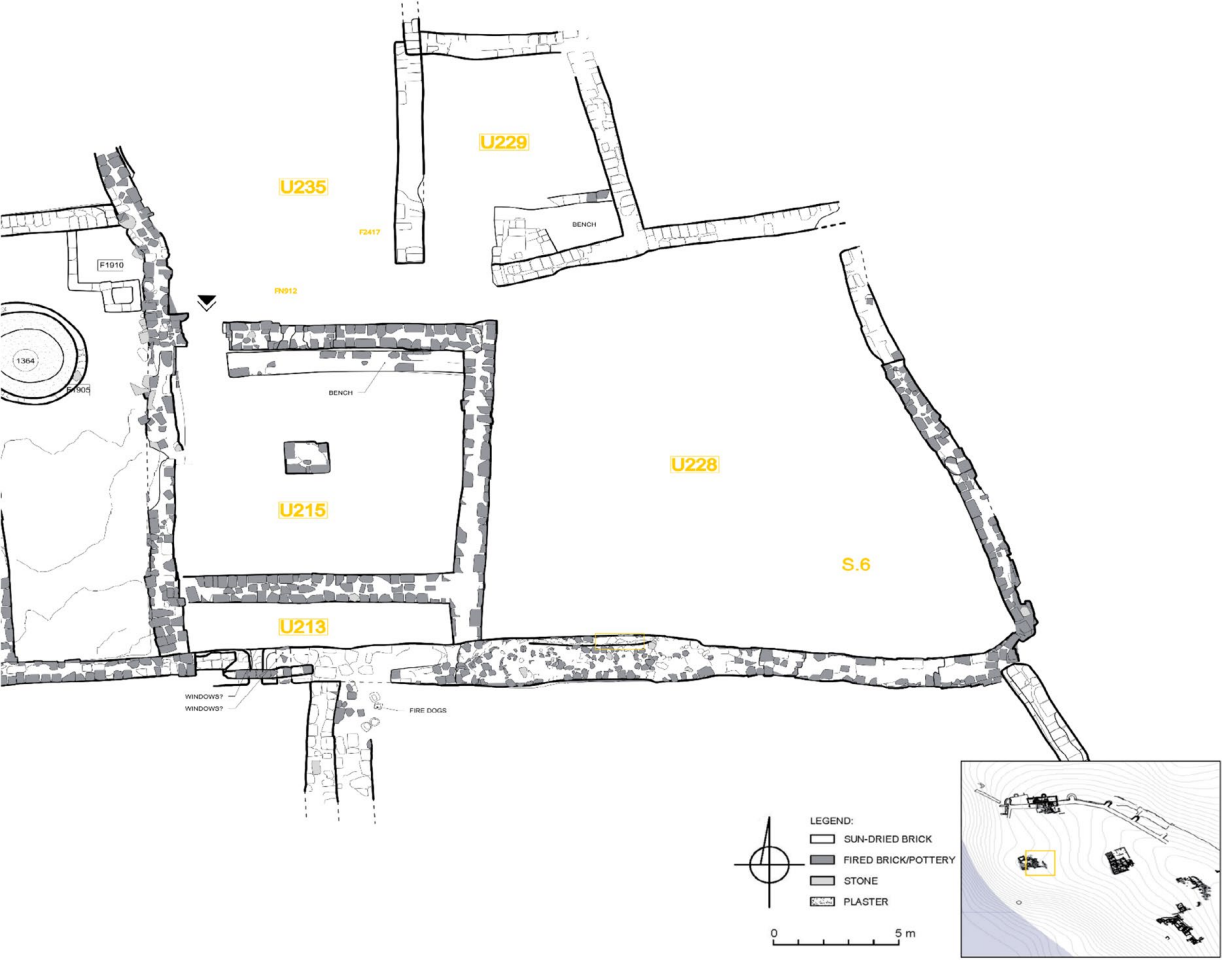
Compound U213/215/228/229/235/245 (drawing: A. Wujec, A. Chlebowski)

**Fig. 6:**
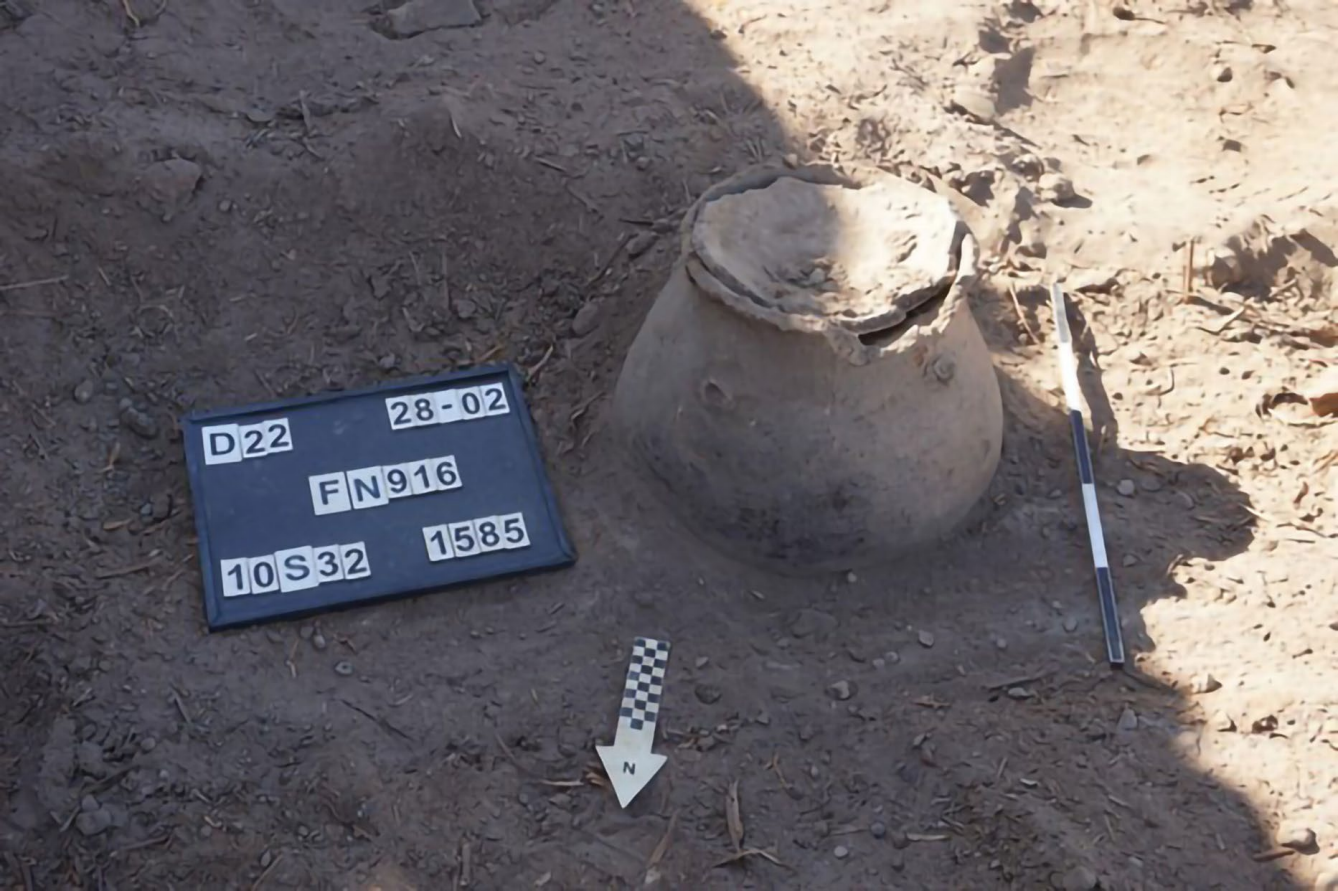
Vessel FN916 (photo: L. de Lellis)

All archaeobotanical samples discussed in this paper were collected from two houses at the citadel of Old Dongola during the excavations in winter 2021–2022. The contents of each storage vessel or basket were carefully emptied into separate plastic bags to maintain the integrity of individual samples. This ensured that each sample represented the entire contents of the respective vessel or basket. The volumes recorded in Table 2 reflect these complete contents. Six botanical samples were collected from three archaeological contexts: five samples were collected from Room U192 (Figs. 2, 3, and 4), while another sample was collected from House U228 (Figs. 2, 5, and 6). To separate fine dust and ash residues from the preserved macrobotanical materials, the samples underwent a dry sieving process using a sequence of mesh sizes: 1 mm, 0.5 mm, and 0.2 mm. The samples selected for this study were chosen specifically for their direct botanical evidence of stored crops preserved in situ, which provided valuable insights into the household agricultural economy and small-scale storage practices at Old Dongola.

**Table 2:** Identified crops that probably derive from storage assemblages at Old Dongola. The table provides a classification of different ways the identified crops and other species could have arrived in the storage containers. This follows the four-tier classification defined by Hillman (1981, 1984) and adopted by de Vartavan (1990)

Unit	U192										U228	
Period	Mid-sixteenth century CE										Fifteenth century CE	
Sample	2184		2097		2099		2100		2101		2853	
Location of the sample	-		-		-		-		-		S6	
Archaeological object type	Vessel		Vessel		Basketry		Vessel		Vessel		Vessel	
Preservation	Charred		Charred		Charred		Charred		Charred		Desiccated	
Volume	1 L		29 L		0.8 L		0.6 L		0.6 L		6 L	
	**Counts**	**Route of arrival**	**Counts**	**Route of arrival**	**Counts**	**Route of arrival**	**Counts**	**Route of arrival**	**Counts**	**Route of arrival**	**Counts**	**Route of arrival**
*Sorghum bicolor* (L.) Moench. Grains	-	-	-	-	-	-	-	-	10	A1*	4879	A1
*Sorghum bicolor* (L.) Moench. Husks/Glumes	70	B1	400	B1	-	-	-	-	-	-	-	-
*Triticum aestivum* L. Grains	2900	A1	4	A1*	30	A1*	-	-	24	A1*	-	-
*Hordeum vulgare* L. Grains	-	-	6	A1*	1	A1 and A6*	11	A1 and A6*	8105	A1	15	B4
*Hordeum vulgare* L. rachis internodes	-	-	-	-	-	-	-	-	-	-	50	B4
*Raphanus raphanistrum* subsp*. sativus* (L.) Schmalh	-	-	2924	A1	-	-	-	-	-	-	-	-
*Citrullus lanatus* (Thunb.) Matsum. & Nakai)	-	-	1	A3 and A6	-	-	-	-	-	-	22	A3 and A6
*Lathyrus sativus* L	-	-	60	A1*	1682	A1	-	-	10	A1*	-	-
*Vigna unguiculata* L	-	-	320	A1*	-	-	8174	A1	4	A1 and A6*	-	-
*Citrullus colocynthis* (L.) Schrad	-	-	1	A3 and A6	-	-	-	-	-	-	2	A3 and A6

Note 1. Codification of possible route of arrival adopted after Hillman (1981, 1984) and de Vartavan (1990). A1: seeds, chaff, grain, etc., arriving as components of crop products. A2: seeds from non-cultivated plants gathered as fodder, bedding, and fuel. A3: seeds from plants gathered as foods, condiments, medicines, or dyes. A4: seeds from plants gathered as “furnishings,” such as rush-matting. A5: seeds derived from peat or dung burned as fuel. A6: seeds arriving casually (i.e., non-systematically). B1: Winnowing waste. (usually light objects). B2: waste from coarse sieving. B3: cleanings from fine sieving—by-products (usually big chaff, such as cereal stalk fragments or large weeds). B4: semi-clean prime grain (from spillage). B5: cleanings for hand-sorting. B6: pure prime grain. C1: twining weeds: these are automatically harvested when the crop is uprooted or reaped either low or medium height on the straw. C2: free-standing weeds: 3/4 height of crop or taller. C3: free-standing weeds: 1/4 to 3/4 height of crop. C4: free-standing weeds: < 1/4 height of crop. D1: weeds of wasteland. D2: pasture or heath species D3: plants of cleared woodland or woodland fringes and glades. D4: marsh and bog species

Note 2. The sign (*) next to the above classifications indicates the in situ contamination, representing the case of in situ contamination while collecting the sample

Note 3. The grams and calories were calculated only for the stored crops

The samples were further processed by sorting and identified using a Euromex stereomicroscope with up to 30 × magnification at the archaeobotany laboratory of the Institute for Archaeological Sciences, University of Tübingen, Germany. Seed identification was conducted through anatomical and morphological comparisons with modern reference specimens from the archaeobotany laboratory at the University of Tübingen. Identification was further supported by seed identification catalogues and atlases (Cappers et al., 2009, 2012; Neef et al., 2012), along with the African Plant Database (https://africanplantdatabase.ch/) to refine taxonomic classification. For the identification of bread wheat (*Triticum aestivum* L.), both grain and chaff remains were examined to ensure reliable identification. The presence of chaff, alongside broader archaeobotanical evidence from sites such as Nauri (Fuller & Edwards, 2001), Qasr Ibrim (Rowley-Conwy, 1989), and Amara West (Ryan, 2018; Ryan et al., 2022), further supports the identification of bread wheat at Old Dongola.

To examine possible depositional mixing of crop storage in archaeological samples, we adopted Gordon Hillman’s ABCD classification system (Hillman, 1981, 1984), which was previously used by de Vartavan (1990) to interpret plant food remains from Tutankhamun’s tomb in Egypt (Table 2) and provides information on how plants may have arrived at the sites (A) and if they represent closed or open contexts (B, D). This system categorized plant remains as follows: (A) mode of arrival of seeds onto the site (the different ways of arrival—A1 to A6—are explained in Table 2, note 1); (B) classification of crops and segetal weeds by the type of crop product or by-product in which each item is normally found; (C) classification of segetal weeds by their growth form and growing height in crop stands; and (D) classification of non-segetal species by the habitat in which they probably grew. Each system contains different sub-categories, as explained in note 1 of Table 2. Furthermore, we considered the potential for in situ contamination during the excavation and sample collection (note 2, Table 2) (Rotchell et al., 2024; van der Veen, 2007). This allows us to determine whether the contaminants were initially deposited in the same context and mixed while excavating the assemblage or whether this resulted from post-depositional processes (de Vartavan, 1990; Hillman, 1984; van der Veen, 2007).

Ethnographic analogies are used in this study to aid in the interpretation of plant use identified in the archaeological record. These analogies were derived from a combination of published botanical literature on Sudanese flora (e.g., Bebawi & Neugebohrn, 1991; Braun et al., 1991; Darbyshire et al., 2015; Harrison & Jackson, 1961; Mubarak et al., 1982), established work on the ancient and contemporary Sudanese cuisine (e.g., Bacon, 1949; Dirar, 1993), and fieldwork conducted in El Ghaddar village (near Old Dongola), where local residents provided insights into the continued use of plants in the traditional cuisine (Nasreldein et al., forthcoming). Ethnographic analogies provide valuable comparative frameworks for interpreting past subsistence practices, particularly in regions where historical continuity in food production and cultural traditions has been observed (e.g., Abdelrahman, 1998; Adams, 1977; Edwards, 2003, 2004; Haaland, 2007; Haaland & Haaland, 2013; Hasan, 1973; Shinnie, 1978). However, their application must be critically assessed, as modern ethnographic data do not always directly reflect past societies, and cultural transformations over time must be considered. As Wylie (1985) has emphasized, while ethnographic analogy remains a useful interpretive tool, it should supplement rather than dictate archaeological interpretations. In this study, such analogies are used with caution, acknowledging both their strengths and limitations in reconstructing past plant use. As Wylie (1985) discusses, concerns about subjectivity in ethnographic analogy remain valid, particularly as Thompson (1958) has argued that archaeological interpretation is inherently interpretative. However, by ensuring a diverse and well-documented dataset, including firsthand fieldwork, we mitigate these concerns and strengthen the applicability of ethnographic comparisons.

### Description of Room U192 (Contexts 1195 and 1199)

House U143/144/160 and the expansive courtyard U73 are the most recognizable compounds in the excavated part of zone 1.1 of the Old Dongola citadel. This compound has been thoroughly examined, starting from its construction and continuing through its abandonment, ultimately transforming the entire area into a waste disposal site (Deptuła & Maślak, 2021). The house was constructed on top of an earlier Makurian structure in the first quarter of the sixteenth century and was in use until at least the turn of the sixteenth and seventeenth centuries CE. The courtyard was continuously inhabited throughout the entire operational phase of the house, and it was successively overbuilt with several superimposed wattle-and-daub structures (U194, U181, U192, U169, U166). These structures were constructed at different stages and were not all used simultaneously.

Building U192 is the only space with a clearly defined function. It was constructed during the same period as House U143/144/160 and was situated in the southwest corner of Courtyard U73. A fire, dated to the mid-sixteenth century (based on radiocarbon dating), caused the wooden roof and walls to collapse, sealing the pantry deposit that was originally stored inside. The space was filled with numerous large containers, two of which (FN853 and FN854) remained in situ, while others were fragmented and mixed with deposits that filled the space. These vessels were used for storing grain. Concentrations of different types of burnt grains and beans were discovered within this unit, and samples of each type were collected for archaeobotanical analysis. One concentration (S2101) was found inside Vessel FN854, covered with a plaited mat, while another (S2099) was surrounded by fragments of burnt basketry, indicating that they were initially stored in such containers. Archaeobotanical samples collected from Contexts 1195 and 1199 comprised mixed materials primarily consisting of fragments of burnt roof and wall structures, fragments of storage vessels, and concentrations of burnt organic materials, which were likely originally contained within the vessels (Fig. 4).

### Description of Compound U213/215/228/229/235/245 (Context 1599)

Residential compound U213/215/228/229/235/245 was located in the north-western sector of the citadel, east of the late-Makurian complex of the building B.VI, previously excavated and interpreted as a storehouse (Danys-Lasek, 2014; Godlewski, 2013; Obłuski, 2014b). This residential compound had a long lifespan, from its first establishment in the fifteenth century to its final demise in favor of a new residential structure in the second half of the seventeenth century. The context we investigated from this house is dated to the second half of the sixteenth century CE. The food storage function is attested by a cooking pot (FN916) reused as a storage vessel and embedded into the matrix of Deposit 1585, located in Courtyard U228 (Fig. 6). A potsherd served as a lid for the vessel and contributed to preserving the original content of stored crops inside the vessel (Context 1599), hereby analyzed.

**Fig. 7:**
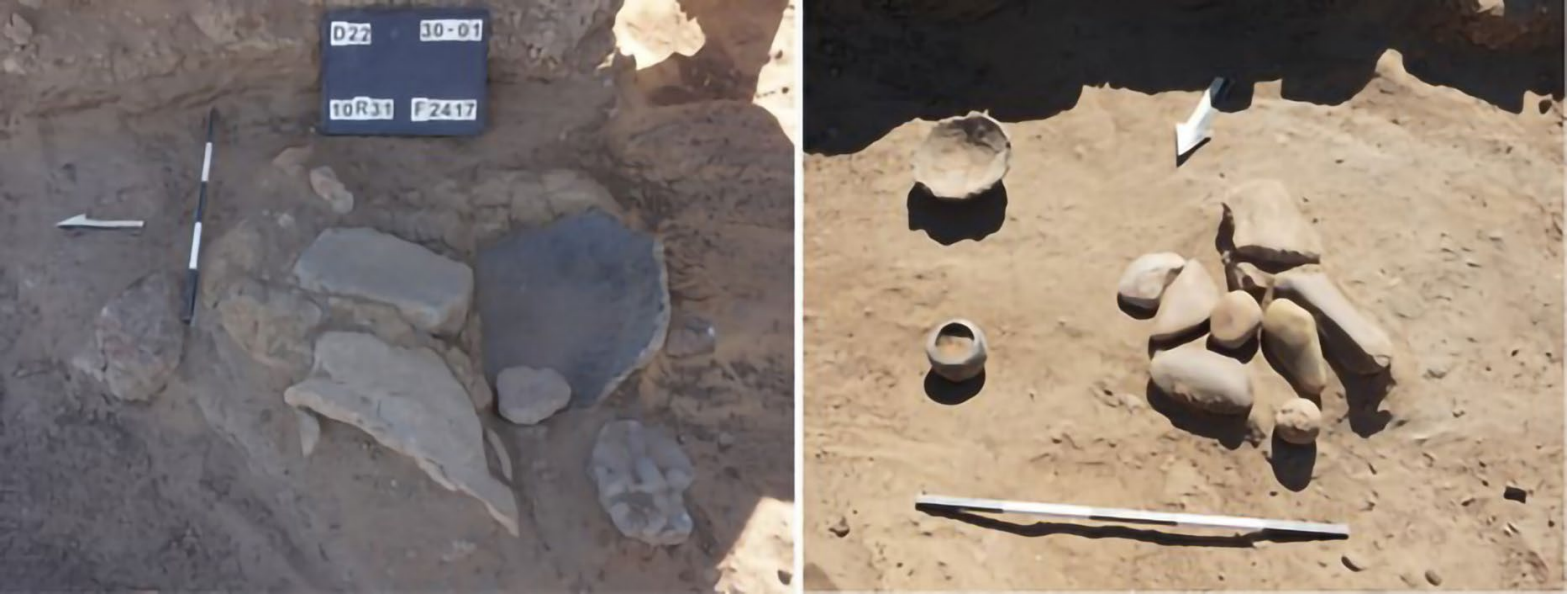
Quern emplacement (F1417) and several grinding stones (FN912) inside U235 (photo: L. de Lellis)

In the first half of the sixteenth century, the area of the compound was organized into a main residential room (U215) flanked by a narrow storage space (U245). A secondary residential unit (U235) abutted U215/245 to the north. Both units were accessed from a shared courtyard, U228. In the second half of the sixteenth century, the compound underwent a reorganization that involved a substantial renovation of the perimeter walls of U215, with the absorption of the space of U245 into the area of U235, which lost its residential function and was transformed into a food preparation area. This interpretation is supported by the recovery still in situ of a quern installation (F1417) and several grinding stones (FN912, Fig. 7). The vast courtyard (U228) primarily served domestic activities, storage, food processing, and potentially animal husbandry, as indicated by the presence of dung.

## Results

**Fig. 8:**
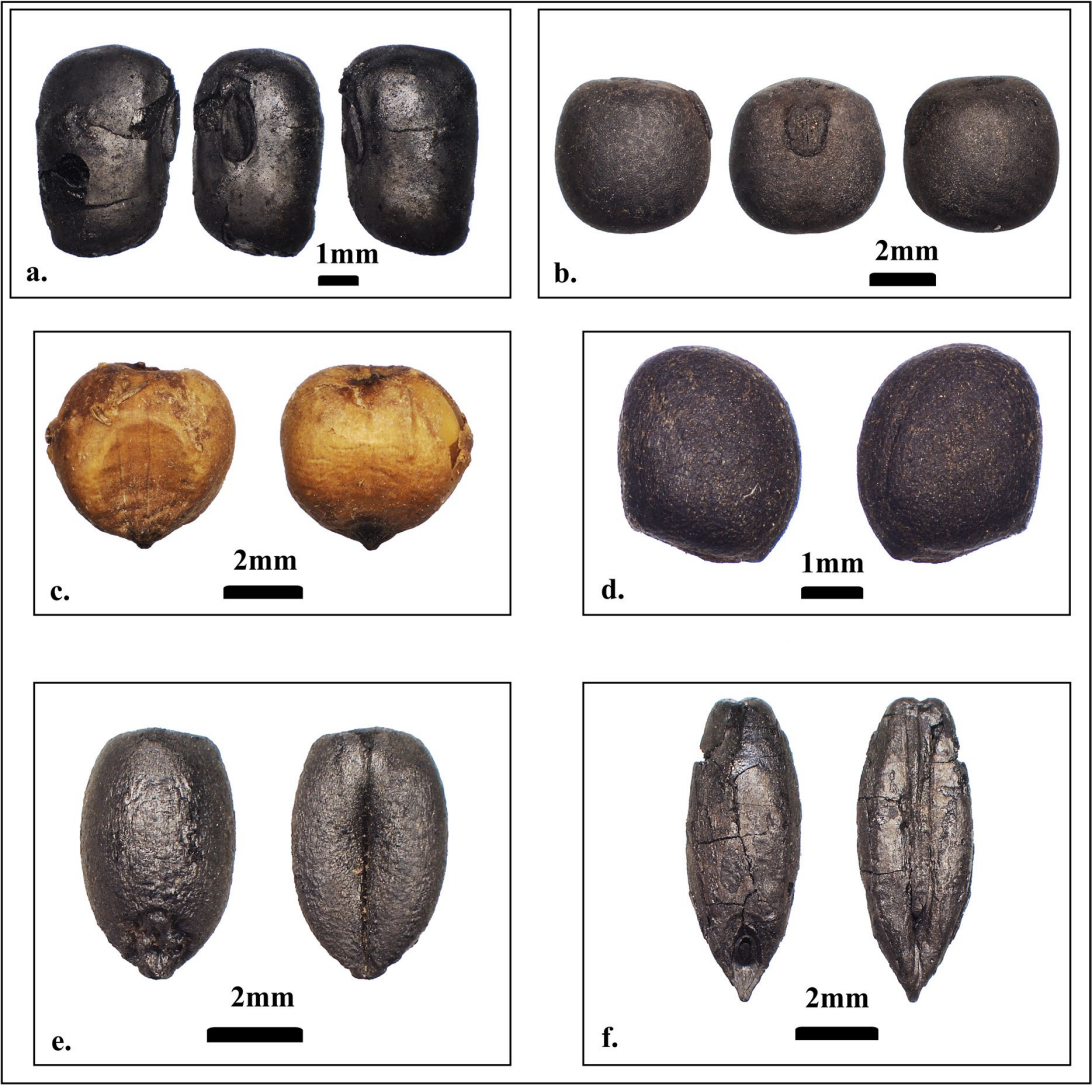
Stored crops at Old Dongola. **a** Cowpea (*Vigna unguiculata* L.), **b** Grass pea (*Lathyrus sativus* L.) from context 1195 U192, **c** Sorghum (*Sorghum bicolor* (L.) Moench.), uncarbonized from context 1599, **d** Radish (*Raphanus raphanistrum* subsp. *sativus* (L.) Schmalh) from context 1199 U192, **e** Bread wheat (*Triticum aestivum* L.), **f** Barley (*Hordeum vulgare* L.) from context 1195 U192. (Photos: Mohammed Nasreldein)

A total of 29,185 seeds and grains, along with 520 chaff fragments, were identified from the storage assemblages discovered in the two houses at Old Dongola. This amounts to a combined total of 29,705 plant remains, representing nine taxa (Table 2), of which seven are cultivated crops. Cowpea (*Vigna unguiculata* L. Walp) is the most abundant crop, representing 29% of the total assemblage, followed by hulled barley (*Hordeum vulgare* L.) representing 28%, sorghum (*Sorghum bicolor* (L.) Moench.) 17%, bread wheat (*Triticum aestivum* L.) 10%, radish seeds (*Raphanus raphanistrum* subsp. *sativus* (L.) Schmalh) 10%, and grass peas (*Lathyrus sativus* L.) representing 6% of the assemblage (Fig. 8).

The majority of crops were stored in different vessels and basketry (see Table 2), discovered in situ, and preserved by charring. In contrast, sorghum grains from U228 were stored in a vessel (context 1599) and preserved by desiccation, containing a mixture of husked and unhusked grains of sorghum, possibly indicating storage in a semi-processed form. Along with sorghum grains, the vessel contained other seed remains in very low quantities compared to the sorghum grains. These crops represented hulled barley (*Hordeum vulgare* L.), watermelon seeds (*Citrullus lanatus* (Thunb.) Matsum. & Nakai), and colocynth seed remains (*Citrullus colocynthis* (L.) Schrad.) (see Table 2).

### Disentangling Crop Storage Contexts: Consumption, Sowing, and Post-Depositional Factors

The two storage contexts examined in this study, U192 and U228, provide insights into household food storage practices at Old Dongola, reflecting different strategies for preserving crops and managing agricultural resources. U192, a pantry within a domestic space, was sealed by a mid-sixteenth-century fire, which caused the collapse of the wooden roof and walls, effectively preserving large concentrations of burnt grains and legumes. The presence of large storage containers, some remaining in situ while others were fragmented and mixed with burnt debris, attests to the deliberate storage of crops in this space. The fire event appears to have sealed the deposits rapidly, preserving assemblages that were in use at the time of destruction.

In contrast, U228 represents an open courtyard setting with a more dynamic use history. The storage sample analyzed from this unit, retrieved from context 1599, was found inside a cooking pot (FN916, Fig. 6) that had been repurposed as a storage vessel and embedded into the courtyard matrix. A potsherd served as a lid, contributing to the preservation of its contents, which consisted primarily of desiccated sorghum grains. The surrounding courtyard space contained installations related to food processing, including a quern installation (F1417) and several grinding stones (Fig. 6), suggesting an integrated system of storage and processing. The function of this assemblage is less straightforward than U192, as the presence of semi-cleaned sorghum grains raises the question of whether these grains were stored for human consumption, longer-term preservation, or future sowing. The practice of storing unhusked sorghum may reflect a deliberate strategy to extend preservation or maintain the viability of grains for human consumption after husk removal or using them for animal feed. In Sudan, fermentation of sorghum products is typical in certain vessels; however, in this situation, we can exclude this possibility because fermentation typically occurs after the sorghum has been processed into dough.

The differentiation between these storage contexts is further complicated by contamination patterns and depositional/post depositional processes. To assess these factors, Hillman’s (1984) ABCD classification system was applied, identifying different routes of contamination. The results in Table 2 show that the classification effectively differentiates the arrival routes within the storage context. For example, desiccated sorghum husks in samples 2184 and 2097 were classified as B1, suggesting they were deposited as winnowing waste, while grains of bread wheat and hulled barley in sample 2853 were classified as B4, indicative of semi-clean prime grains from spillage. Additionally, the classification system identified other contaminants ranging from A1 to A6, providing a clearer picture of the potential arrival routes to the storage context, as detailed in Table 2, note 1. Therefore, this classification offered a definitive framework for identifying sources of contamination and the potential arrival routes of plant remains in archaeological contexts.

In U192, contamination primarily resulted from pre-depositional mixing and in situ processing activities. The assemblage from sample 2101, which mainly contained hulled barley, also included charred remains of bread wheat, cowpea, and grass pea, suggesting that crops were stored in proximity and mixed before final deposition. Notably, the only charred sorghum grains found in the assemblage were within this sample, implying that these grains were incorporated into the deposit during routine household activities rather than being originally stored in the pantry.

In U228, contamination patterns suggest a different sequence of depositional events. Sample 2853, consisting of desiccated sorghum grains, contained contaminant remains of desiccated hulled barley (grains, rachis, and spikelets), along with watermelon and colocynth seeds. Unlike the situation in U192, where contamination largely occurred before deposition, the contamination in U228 appears to be related to vessel reuse, wind-blown deposition, and post-depositional mixing. The presence of hulled barley in the sorghum storage sample indicates that the vessel had previously been used for barley before being repurposed for sorghum, with residual grains remaining in the container. Additionally, the presence of desiccated colocynth and watermelon seeds suggests later contamination, possibly due to exposure in an open environment or the accumulation of domestic refuse over time. The sequence of contamination in U228, therefore, highlights the more exposed and dynamic nature of courtyard storage, in contrast to the sealed pantry environment of U192.

A broader comparison with archaeobotanical evidence from Old Dongola further contextualizes these findings. Recent study (Wyżgoł et al., 2024) has demonstrated that household processing of staple crops played a central role in the courtyards at Old Dongola. The presence of grinding stones in U228 supports the interpretation that the stored grains were probably intended for gradual processing into flour. This contrasts with U192, where the evidence suggests a storage space dedicated more strictly to bulk crop preservation rather than active processing. The findings from these two units thus reflect not only different spatial storage strategies but also the complexity of food preservation and preparation practices in the Kingdom of Dongola and the Funj period.

Moreover, our findings at Old Dongola align with earlier evidence of grain storage practices across Sudan, as summarized in Table 1. The use of jars (*gossi*/*gossiba*) persisted across Northern Sudan for millennia, supporting the interpretation that the observed storage patterns at Old Dongola are part of a broader Sudanese traditions. However, as previous studies have shown (e.g., Bonnet, 1992; Garcea & Hildebrand, 2009; Hildebrand & Schilling, 2016; Honegger, 2003, 2004; Tahir et al., 2015; Welsby, 2008; Wolf et al., 2014) detailed insights into storage variables such as vessel capacity, spatial distribution, and specific crop identifications remain limited, reinforcing the significance of our study in addressing these gaps.

## Discussion

### Crops of African Origin Arriving from West, Central, or Eastern Africa

The group of crops of African origin, referred to as the “Savannah package” by Fuller and Lucas (2020: 936), is characterized by its high drought resistance, as noted by Clapham (2019: 85). Archaeological evidence suggests that these crops have been present in Sudan since the early Holocene, as indicated by the discovery of wild sorghum and various millet types, including *Panicum* sp., *Echinochloa* sp., *Setaria* sp., and *Brachiaria* sp., alongside pearl millet (A-Magid, 2003; Beldados et al., 2015; Fuller & Lucas, 2020: 936; Haaland, 1995, 1999; Haaland & Haaland, 2013; Stemler, 1990). The spread of these crops into Sudan and further northward to Egypt is likely linked to regional interactions and movement along the Nile River system (Clapham, 2019: 85).

#### Cowpea (*Vigna unguiculata* L.)

Cowpea is a summer-grown crop native to the savannah of West Africa (Allaby et al., 2008; D’Andrea et al., 2007; Larson et al., 2014; Panzeri et al., 2022). It was probably first brought under cultivation 2000 years ago (Champion et al., 2021; D’Andrea et al., 2007; Vaillancourt & Weeden, 1992). Genetic studies suggest a single origin of domestication that is as yet undetermined (Herniter et al., 2020; Muñoz-Amatriaín et al., 2021; Sarr et al., 2021), most probably in Ghana from the Kintampo B-sites dating back to 1830–1595 BC (D’Andrea et al., 2007; Neumann et al., 2022). Panzeri et al. (2022) highlighted cowpea as one of the world’s most essential legumes, representing significant social and economic value in many African countries, mainly where animal proteins are unaffordable for the local population. Numerous studies have underscored the nutritional value of cowpea seeds, which are abundant in essential amino acids and have high levels of minerals, lipids, and vitamins (Boukar et al., 2011; Duranti, 2006; Harouna et al., 2018; Marconi et al., 1997).

The earliest evidence of pulses in Sudan comes from the New Kingdom Egyptian town of Amara West (Ryan et al., 2012). Fuller and Lucas (2020: 935) have argued that there might be a gap or lack of evidence for the crop’s earlier presence due to limited systematic sampling. Thus, gaps in the archaeobotanical record—particularly regarding legumes—may reflect sampling limitations rather than the actual absence of these crops in historical diets. However, besides the pulse crop remains from Amara West, other evidence of cowpea consumption can be found during the Meroitic period at Mouweis in central Sudan, dated 100–300 CE (Bouchaud et al., 2018: 380), and further evidence dating to the Christian and Early Islamic periods was provided by Qasr Ibrim (Clapham, 2019: 88; Clapham & Rowley-Conwy, 2007: 159–162). Cowpeas were also common in the Alwan diet (fifth to fifteenth centuries CE) at Soba in Central Sudan (Cartwright, 1998: 255–268; van der Veen & Lawrence, 1991: 267).

Some Arab travelers reported peas as part of the staple diet at Old Dongola during the Makurian period. Al-Omari noted that in *c*. 1348, the Nubians at Old Dongola consumed meat, milk, and fish. The best of their foods was prepared from peas in meat extract poured on porridge (Al-Omari, 2010: 49). Dirar (1993: 10) argued that the peas described by Al-Omari are cowpea. A recent study by Ryan et al. (2022) reported cowpea as an important pulse crop for the Nubians, as they grew cowpea for their subsistence and as fodder crops for their household sheep, goats, and cattle.

Cowpea, locally known as *lubia helu*, is a staple in Sudanese agriculture, commonly cultivated in irrigated or river-flooded areas throughout the year. According to Bacon (1949: 349), it comes in two main varieties—small with dark green foliage, short erect pods, and tiny brownish seeds, and large with spreading plants, long pendant pods, and large wrinkled white seeds. The leaves are used in salads or stew *mullah*, and the smaller variety’s immature pods serve as a valuable summer vegetable (see Ryan et al., 2022). Cowpea seeds are often boiled *belila* or roasted and eaten occasionally or during Ramadan in present-day Nubia (Bacon, 1949: 349; Ryan et al., 2022).

#### Sorghum (Sorghum bicolor (L.) Moench.)

Sorghum, native to Africa (Fuller & Stevens, 2018: 427), has historically played a key role in agriculture, serving multiple purposes, including human consumption, animal feed, and, more recently, ethanol production (Kasegn et al., 2023). The domestication and early cultivation of sorghum in Sudan remain subjects of scholarly debate (Beldados, 2018: 503–528; Fuller & Stevens, 2018: 427–452; Rowley-Conwy et al., 1999: 55–61; Winchell et al., 2017: 673–683). The earliest evidence of wild sorghum in Sudan comes from Neolithic sites in Central Sudan, dating to around 6000 BC (Haaland, 1999: 399; Winchell et al., 2017: 673). On the other hand, the evidence for domesticated sorghum appears later, around 3000 BC in Eastern Sudan (Fuller & Stevens, 2018: 427; Winchell et al., 2017: 673).

At Old Dongola, sorghum is the most dominant cereal crop, yet there is no direct evidence of its cultivation before the fourteenth century (Nasreldein et al., forthcoming). The archaeological evidence indicate that sorghum had played a significant role in Sudanese agriculture, which expanded significantly between the late Christian and Ottoman periods (1200–1800 CE), as evidenced by increased finds at Qasr Ibrim (Rowley-Conwy, 1989: 134), suggesting a parallel trend at Old Dongola.

The historical presence of sorghum-based foods in Sudan can be traced archaeologically. One of the most distinguished sorghum-based products is *kisra*, a staple Sudanese flatbread made with fermented sorghum dough and heated on ceramic griddles known as *doka* plates. *kisra* baking using *doka* plates was evident at the Meroitic townsite of Hamadab (300 BC to 350 CE), where archaeobotanical investigations confirmed that sorghum was the primary cereal crop (Fuller & Carretero, 2018: 115–116; Matthews & Nowotnick, 2019; Wolf et al., 2014). A similar bread-making tradition is evident at Old Dongola, where *doka* plates were frequently found near grain-grinding areas and fireplaces—usually three stone hearths (Danys, 2022: 119; Wyżgoł, 2022: 145). Historical accounts also reference daily baking of *kisra* in Nubia (Burckhardt, 1819: 219). During the Funj period, a shift toward *doka* plate dominance for flatbread-making may be linked to politico-religious changes (Edwards, 2003: 147).

Additionally, the tradition of consuming sorghum porridge (*asida*) in Sudan was previously documented from the Neolithic through the Meroitic period (Edwards, 2003: 143–144; Haaland, 2007: 177). Recent archaeological evidence further strengthens the importance of sorghum in the Sudanese cuisine. Excavations at the Amun temple at Dangeil uncovered ceramic (bread molds) containing remnants of porridge that had been shaped into conical loaves, indicating that a porridge-like sorghum product—possibly resembling *asida—*may have been stored or offered in ceremonial contexts (Anderson & Ahmed, 2006; Anderson et al., 2007). This discovery suggests that sorghum played an integral role in both domestic food practices and ritual offerings. Furthermore, the adoption of bread molds for sorghum-based products at Dangeil mirrors broader adaptive strategies seen in other African societies. For instance, in Ethiopia, indigenous grains such as tef and millet were adapted to griddle baking, despite the presence of Near Eastern cereals like wheat and barley (Lyons & D’Andrea, 2008: 525). This example highlights how communities often modified imported baking technologies to suit their available resources and culinary traditions. Similarly, the use of Egyptian-style bread molds at Dangeil likely reflects a localized adaptation of established baking methods, combining elements of Egyptian ceremonial practices with the reliance of Sudanese communities on sorghum as a staple crop.

Complementing this evidence, textual source written in the Egyptian hieroglyphs discovered at Sanam temple from the reign of the Kushite king Taharqa (690–664 BC), the Sanam Historical Inscription further emphasize the cultural significance of sorghum-based foods (Pope, 2013). The inscription references Nubians as eaters of *iwesh*, described as a sticky or gummy food which interpreted as a sorghum product, the term *iwesh* is notably linked to offerings brought to temples in narrow-necked globular jars, suggesting that sorghum porridge or a similar product may have held symbolic value alongside bread loaves, grapes, and dates (Pope, 2013).

Although *kisra* and *asida* are the only sorghum-based products directly identified at Old Dongola due to the presence of *doka* plates and the ceramic bowls, the dominance of sorghum in the plant assemblage and its established cultural significance in Sudanese cuisine strongly suggest it was an essential component of the Funj period diet. The absence of direct evidence for other sorghum products may reflect preservation biases rather than a lack of dietary diversity. The combined archaeological, historical, and textual evidence highlight the long-standing importance of sorghum in both domestic and ritual contexts in Sudan.

The linguistic evidence reveal some contradictions in the origin of the words *kisra* and *asida*, for instance, Dirar (1993: 169) argued that *kisra* is an old Arabic word (Al-Tayib, 1964), and together with *asida* were mentioned in early Arabic literature (Akel, 2014). However, Osman (1978: 103) stated that *kisra* is not common in present-day Nubia (even though the name *kisra* originated in the Nubian language). However, archaeological materials and historical texts strongly suggest that *kisra* remained a significant element in Nubian cuisine.

#### Sorghum in Modern Sudanese Cuisine

The use of ethnographic analogy provides additional insights into possible plant uses in historical Sudanese contexts. As mentioned in the “Materials and Methods” section, these analogies are derived from botanical literature, historical sources, and fieldwork conducted in the Old Dongola region, with a critical approach that acknowledges their interpretive limitations (Wylie, 1985). Sorghum, locally known as *dura* in Sudan, remains central to contemporary Sudanese cuisine, where it is locally known as *dura* and serves as the primary staple food crop (Abdelrahman, 1998). Unlike European breads, Sudanese sorghum-based breads are typically non-spongy and made from fermented or unfermented dough, referred to as *ajin* (Dirar, 1993: 168). Sudanese culinary traditions include 11 distinct sorghum bread varieties (Dirar, 1993: 168) (Table 3). The porridge-based dish *asida* or *lugma*, widely consumed today, is also historically documented from the Neolithic through the Meroitic period (Edwards, 2003: 143–144; Haaland, 2007: 177). Sorghum-based drinks, such as *abri* (or *helu-mur*) and *marisa* (or *bouza*), have long histories but lack archaeological evidence (Bacon, 1949: 315; Dirar, 1993: 187; Cailliaud, 1826: 110–115).

**Table 3:** Summary of the key dishes and items related to sorghum in Sudanese cuisine, along with brief descriptions and relevant sources

Dish/item	Description	Artifacts at Old Dongola	Source(s)
*kisra*	Thin flatbread made from fermented sorghum dough and often enjoyed with spiced stew (*mulah*)	Ceramic baking plates (*doka*)	(Bacon, 1949: 315; Burckhardt, 1819: 220; Danys, 2022: 119; Dirar, 1993: 168)
*asida*/ *lugma*	Sorghum flour dough is cooked into a porridge, sometimes with additional ingredients	Ceramic and wooden bowls	(Bacon, 1949: 315; Dirar, 1993; Edwards, 2003; Haaland, 2007 Cervi, 2022: 233; Danys, 2022: 61)
*abri/* *helu-mur*	Non-alcoholic beverage by soaking dried *kisra-*like shape in water and often consumed during Ramadan. The preparation begins before Ramadan due its multi-step process. Sorghum is soaked, sprouted, sun-dried, and grounded into flour. The flour is then mixed with hot water and several spices, including cinnamon, ginger, cardamon, and hibiscus. The mixture left to ferment and then cooked in a similar way to *kisra* in flat plates like *doka* or *saj*	No direct evidence, only possible inference from ceramic baking plates (*doka*)	(Bacon, 1949: 315; Dirar, 1993: 187)
*marisa*/*bouza*	African opaque beers made from sorghum, ranging from thin porridge to dark beer	Drinking cups, beaker, and the ceramic jars	(Bacon, 1949: 315; Cailliaud, 1826; Cervi, 2022: 233; Danys, 2022: 61; Dirar, 1993: 224)
*aragi*	Potent spirit distilled from high-alcohol content and considered illegal today	No direct evidence	(Bacon, 1949: 315; Dirar, 1993)
Sorghum grains	Consumed boiled (*belila*) or roasted (*galeia*), it is also used as feed for poultry and livestock	Bowls, ceramic and wooden plates for serving food	(Bacon, 1949: 315; Cervi, 2022: 233; Danys, 2022: 61)

Another significant dish in Sudanese cuisine is *asida* or *lugma*, which is a sorghum flour dough prepared similarly to *kisra* but cooked into a porridge, often with additional ingredients. *lugma* is a similar but less superior preparation (Bacon, 1949: 315). Furthermore, sorghum is widely utilized in the production of *abri* or *helu-mur*, meaning sweet-bitter drink, which is a thirst-quenching, non-alcoholic drink made by soaking dried *kisra*-shape flakes in water, usually prepared for breakfast during the holy month of Ramadan (Bacon, 1949: 315; Dirar, 1993: 187). Although this product is significant within the Sudanese community, its origins remain unknown due to the lack of historical records that could elaborate on its past.

Sorghum is also a key ingredient in the preparation of alcoholic beverages, such as *marisa* or *bouz*a (Cailliaud, 1826: 110–115), which is a member of the African opaque beers, ranging from a slightly fermented thin porridge to a dark beer with an alcohol content of up to 4 or 5% (Bacon, 1949: 315; Burckhardt, 1819; Dirar, 1993: 224; Edwards, 2003: 143–144; Haaland, 2007: 166). In certain regions of Sudan, men primarily consume *dura* in the form of *marisa*; according to Dirar (1993: 225), the bulk of *marisa* is homemade and is consumed within the family circle. Moreover, sorghum contributes to the production of *aragi* a potent spirit forbidden by law and obtained through the distillation of high-alcohol content *marisa*. The word *aragi* is also used for the spirit distilled from fermented date liquor (Bacon, 1949: 315). Besides those mentioned above, the historical and ethnographic records do not reveal any further additional uses of sorghum in Sudan.

### Crops with a Mediterranean and SW Asian Origin

The agricultural practices of early Egypt were heavily influenced by Middle Eastern crops such as wheat, barley, pulses, and flax (Bellwood, 2023; Fuller & Lucas, 2020; Murray, 2000a, 2000b; Zohary et al., 2012). These crops, used from the Neolithic period in western Asia, were integral to Egyptian agriculture, spreading from Lower Egypt up the Nile into Nubia (Clapham, 2019: 83–101; Fuller & Lucas, 2020: 927–947; Haaland, 2007: 165–82; Ryan et al., 2012; Wetterstrom, 1998: 165–226). Recent discoveries suggest wheat and barley cultivation in Middle Nubia as early as 5000 BCE (Madella et al., 2014; Out et al., 2016). The introduction of these crops marked significant milestones in developing agricultural societies along the Nile Valley (Clapham, 2019: 83; Fuller, 2015: 33; Fuller & Lucas, 2020: 928; Ryan et al., 2012).

#### Bread Wheat (*Triticum aestivum *L.) and Hulled Barley (*Hordeum vulgare* L.)

Bread wheat and hulled barley are important winter crops representing staple cereals at Old Dongola (Nasreldein et al., forthcoming). Initially domesticated in the Levantine region of Southwest Asia over 10,000 years ago, these crops became established in the Egyptian Nile Valley by 5000 BCE (Bellwood, 2023; Fuller & Lucas, 2020: 933; Zohary et al., 2012; Zohary & Hopf, 2000). The first evidence of emmer wheat in Sudan dates back to the Neolithic period in the Middle Nile Valley (A-Magid, 2003; Haaland, 1999; Haaland & Haaland, 2013), while North Sudan shows wheat and barley cultivation by the fifth millennium BCE (Hildebrand, 2007; Hildebrand & Schilling, 2016; Madella et al., 2014; Out et al., 2016; Reinold, 2000). Several authors (see Edwards, 2003: 137; Fuller & Lucas, 2020: 927; Haaland, 2007: 165; Haaland & Haaland, 2013: 537; Ryan et al., 2012) have intensively studied the dispersal of these Near Eastern crops through Egypt into Nubia. Their findings confirm that wheat and barley played a significant role in food culture and diet, integrating Mediterranean influences into Nubian cuisine.

The tradition of baking leavened wheat-based bread in ovens, which originated in the Near East and spread into Egypt, became part of Nubian culinary traditions (Edwards, 2003:137; Fuller & Carretero, 2018: 115; Haaland, 2007: 165). Domed ovens (box ovens) were introduced in Nubia, likely as an adaptation of Near Eastern baking methods (Maillot, 2016). These ovens, discovered in Egyptian New Kingdom sites in Nubia such as Amara West, indicate that baking wheat bread was a well-established practice (Spencer et al., 2014). However, historical evidence of bread ovens in Sudan is scarce, particularly in Old Dongola. To date, only one domed bread oven has been discovered, in the Monastery on Kom H, outside the citadel, dating to the Makurian period (Dzierzbicka & Deptuła, 2019: 89). Because of this limited evidence, it remains uncertain whether a major transition in bread-making practices occurred between the Makurian and Funj periods. So far, the available archaeological evidence from the Kingdom of Dongola and the Funj period (fourteenth to eighteenth centuries CE) indicates that ovens are entirely absent from the record with intensive presence of *doka* plates (Danys, 2022: 119–127). This evidence indicates clearly a shift from leavened, oven-baked bread to flatbread-making using griddles (*doka* plates).

This observation aligns with historical accounts, which refer to the daily baking of *kisra* in Nubia (Burckhardt, 1819: 219). Edwards (2003: 147) attributes this shift in bread-making traditions during the Funj period to politico-religious changes in the region. The shift from oven-baked bread to griddle-based flatbreads during the Funj period aligns with broader patterns observed in other African societies, where griddles were widely adopted to bake breads from starchy foods lacking gluten (Lyons and D’Andrea, 2008). In Ethiopia, for example, indigenous grains such as tef and millet were adapted to griddle baking, even though Near Eastern cereals like wheat and barley were present (Lyons & D’Andrea, 2008: 525). This suggests that baking technologies are closely tied to existing sociotechnical systems rather than determined solely by the type of grain available.

Given this, the presence of wheat and barley at Old Dongola in the Funj period without associated ovens may indicate their use in foods suited to griddles rather than ovens. Similar to Sudanese dishes today, wheat and barley could have been prepared as *gurassa* (a thick, spongy flatbread), *fateer* (a wheat- or barley-based flatbread similar to *kisra*), or *madida* (a porridge-like dish combining wheat flour with fenugreek). Additionally, these grains may have been used in various boiled porridges, which are common in Sudanese cuisine. This adaptation highlights the flexibility of food traditions, where local practices shaped how these crops were integrated into the culinary customs. Additionally, the absence of ovens in the Funj period and the prevalence of *doka* plates suggest a growing reliance on sorghum-based flatbreads, replacing wheat-based loaves.

Furthermore, the increasing prominence of sorghum products (*kisra* and *asida*) over wheat bread aligns with historical accounts of shifting agricultural practices and changes in crop cultivation (Nasreldein et al., forthcoming). The Funj period saw a growing emphasis on indigenous African crops, including sorghum, millet, and pulses, which may have been more accessible and better suited to environmental conditions than wheat and barley. This transition likely reflects broader environmental transformations, as proxy records indicate a shift toward increasingly drier conditions in the Middle Nile Valley after the first millennium BC (Machado et al., 1998; Williams, 2009: 10—11). Isotopic data from human and faunal remains further support this shift, showing increased reliance on C4 plants like sorghum and millet—better adapted to arid environments—during the post-Meroitic period (350–500 CE) and the later periods (Ciesielska et al., 2024; Kozieradzka-Ogunmakin & Sołtysiak, 2023). This transition suggests a reorientation of food culture away from Near Eastern influences and toward a more localized Sudanese dietary tradition.

Early Arab travellers and classical writers noted wheat and barley as key food crops in medieval Nubia, reflecting their importance in diet and trade (Al-Omari, 2010: 49; Al-Qazwini, 1960: 39; Maqrizi, 1270: 190). However, by the later Funj period, oral histories from the Old Dongola region suggest that wheat was cultivated only in small quantities for domestic use rather than commercial purposes. Given that wheat is not drought-tolerant, this reduction in cultivation may reflect broader environmental pressures—particularly increasing aridity—which made wheat less viable as a staple crop in the region. This pattern aligns with paleoenvironmental studies that document increasing dryness in the Middle Nile Valley after the first millennium BC (Kozieradzka-Ogunmakin & Sołtysiak, 2023; Machado et al., 1998; Williams, 2009: 10—11). It is likely that, during periods of agricultural scarcity, Nubians turned to the village trade system (barter) to cover the shortage and secure their necessities, with villagers exchanging goods (dates, wheat, and pulses) at larger trading centres (Osman, 1978: 101). Indicating that barter trade and local commerce became a common practice, where wheat was exchanged for other staple crops such as pearl millet.

Additionally, wheat and barley were deeply embedded in Nubian cultural practices. Osman (1978: 101) describes how wheat-based foods played an important role in marriage ceremonies and funerary rituals, while barley was used for animal feed and its straws for building materials. Despite the decline in wheat cultivation, its symbolic value in Nubian society persisted. This symbolic importance may reflect the rarity of these crops in later periods, when their practical use diminished. Like in many cultures, food items that become scarce often gain heightened social or ritual value and are reserved for special occasions such as feasts, ceremonies, or rites of passage.

Oral narratives from Old Dongola indicate that wheat was primarily used for traditional Nubian dishes, such as “elkarboog,” a stew made from coarsely ground wheat grains, onion, tomato paste, and spices. Additionally, barley was commonly consumed in non-alcoholic beverages, believed to have medicinal qualities (Ryan et al., 2022). Beyond its use in food, wheat and barley had multiple practical functions. Green barley was used to fatten livestock, while grains were fed to horses and donkeys in winter. Barley straw (*tibn*) was also used as cattle fodder and as a construction material for making mud bricks (Bacon, 1949: 322–323).

#### Radishes (*Raphanus raphanistrum *subsp. *sativus* (L.) Schmalh)

The origins of radish domestication remain unclear, as there is no clear archaeobotanical evidence to determine their early history (Kim et al., 2016; Zohary & Hopf, 2000). According to Kim et al. (2016), the first evidence of radish consumption in human nutrition came from the Nile Valley, possibly dating back to 2000 BCE in ancient Egypt. Warwick (2011) suggested many possible centres of origin, including the Middle East, Eastern Mediterranean, and Asian regions. Several studies have suggested that the domestication of wild radishes occurred independently in different regions (Yamagishi, 2004; Yamagishi & Terachi, 2003). Radishes appear in historical texts only in the third century BC, when Greek and Roman agriculturalists reported different radish varieties (Lewis-Jones et al., 1982).

In Nubia, radish seeds were discovered at Qasr Ibrim in contexts dating from the Napatan to the early medieval period but seem to disappear in later contexts (Clapham, 2019: 89; O’Donoghue et al., 1996: 541). Fuller and Edwards (2001) mentioned that radish seeds were absent from medieval contexts at Nauri and are rarely grown in the region today.

Radish, primarily consumed for its roots, is gaining popularity for its leaves and sprouts internationally (Butt et al., 2018; Manivannan et al., 2019; Radovich, 2018). Commonly used in salads, the root can be cooked, salted, or processed into dried or canned pickles (Hadley & Fordham, 2003; Nishio, 2017). Asian cuisines frequently incorporate radish pickles (Manivannan et al., 2019; Nishio, 2017). Leaves and sprouts are consumed raw in salads (Gamba et al., 2021).

In modern Sudan, radish is known locally as *figl* and is cultivated in almost every garden. It is commonly found along minor irrigation divisions and in the spaces between other crops. The leaves, seed pods, and roots are frequently consumed (Bacon, 1949: 361). However, the discovery of radish seeds in large quantities from the Funj period plant assemblage at Old Dongola contributes to our knowledge of this important crop as a main part of the economic practices in Nubia. Most importantly, radish seeds were found to be stored in situ in a basket, providing a rare example of crops stored in a Nubian site after Qasr Ibrim (see O’Donoghue et al., 1996: 541). This evidence represents seed material for planting next season, indicating that radishes were cultivated in the region.

#### Grass Pea (*Lathyrus sativus* L.)

Grass pea is one of the oldest cultivated crops with a long history of domestication (Zohary et al., 2012). Historically, grass peas were distinguished as an exceptional food reserved for kings, contrasting sharply with its modern association as a staple for poor communities (Lambein et al., 2019). Archaeological excavations have revealed carbonized grass pea seeds from several southwestern Asia, Aegean, and west Mediterranean Neolithic settlements, such as Turkey (Nesbitt & Samuel, 1996; van Zeist, 1972), Iraq, and Iran (Helbaek, 1960). Similarly, seeds from contexts dating to *c.* 2500 BCE were identified in India (Kislev, 1989) and the Balkans from *c.* 8000 BCE (Nesbitt & Samuel, 1996). Funeral offerings discovered within Egyptian pyramids included various legume seeds, including grass peas (de Vartavan, 1990). In addition, the archaeobotanical and textual evidence from Egypt demonstrates that the grass pea (*Lathyrus sativus* L.) was grown deliberately as animal fodder since the Greco-Roman period (Murray, 2000b: 638).

*Lathyrus sativus* L. is a nutrient-dense legume that plays a significant role in food and nutrition security across many low-income and drought-prone regions (Lambein et al., 2019). With a protein content ranging between 18–34% in seeds and 17% in mature leaves, it is a valuable dietary protein source—often higher than field pea, faba bean, and lupine (Lambein et al., 2019). In addition to its resilience and minimal cultivation needs, it is rich in lysine and contains a high proportion of polyunsaturated fatty acids, making it both a practical and nutritious food for human consumption (Lambein et al., 2019). Numerous studies indicate that consuming large quantities of grass pea seeds can lead to Lathyrism, a condition marked by motor system dysfunction due to nonprotein amino acid neurotoxins present in the seeds of the *Lathyrus sativus* L. and similar species (e.g., Ahmad, 1982; Hao et al., 2017; Lambein et al., 2019; Mishra et al., 2013; Singh & Rao, 2013, 2013). Symptoms manifest as painful muscle spasms that can progress to irreversible paralysis of humans’ lower limbs and animals’ hind limbs (Ahmad, 1982; Singh & Rao, 2013). Boiling the grass peas for 2 h can reduce the toxic components by up to 85%, whereas boiling for 1 h eliminates about 70% of the water-soluble neurotoxin, rendering excessive consumption potentially hazardous (Jha, 1987; Zohary et al., 2012).

Likewise, Fuller and Lucas (2020: 934) elaborated on the presence of grass peas in ancient Egypt, suggesting they may have arrived in Nubia through Egypt, with Nubia providing a frontier between the winter-cropping regime of the north and the Savannah regime of the south. Furthermore, Fuller and Lucas (2020: 941) debated the presence of grass peas in Nubia in Middle and New Kingdom Egyptian sites in Sudan (Cartwright, 2001; Ryan et al., 2012), through to the Napatan period (*c*. 900–350 BCE) (Fuller, 2004). Qasr Ibrim has provided evidence of the consumption of grass peas during the early medieval period (Clapham, 2019).

Grass pea, locally known in Sudan as *gilban*, is annually cultivated on a small scale during the winter in the northern province. The foliage and seeds are typically utilized as animal fodder (Bacon, 1949: 353). Ryan et al. (2022) reported that in modern times, at Ernetta Island north of the 3rd Cataract, the grass pea is consumed on a small scale for food and fodder and is usually found growing feral in broad bean fields.

Due to its light irrigation water needs relative to its yield, which can reach approximately half a ton of seeds and an equivalent weight of dry forage after around four months of growth, the seeds are frequently consumed by farmers and poorer communities (Bacon, 1949: 353). At Old Dongola, it appears that during the Funj period, the grass pea played an essential role in the culinary practices, which remains an indicator of its economic value.

While the discovery of cowpea and grass pea at Old Dongola is significant, the limited quantity of finds necessitates caution in claiming these legumes played a major role in the Funj period diet. However, this finding aligns with the observed differences between this case study and the broader range of contexts at Old Dongola. For instance, Nasreldein et al. (forthcoming) identified sorghum, wheat, and barley as the primary crops at Old Dongola. As discussed, these differences may stem from sampling conditions, preservation biases, or variations in household storage practices. Together, both studies contribute complementary insights into the complexities of crop use at Old Dongola. The above historical and ethnographic significance of legumes in Sudanese cuisine further suggests they may have contributed to local food practices, albeit at an uncertain scale.

### Possible Dietary Preferences

While precise calorific estimations are challenging due to limited data, the presence of sorghum, wheat, and barley in significant quantities suggests that these cereals were likely staple carbohydrate sources during the Funj period. Their dominance in the archaeobotanical assemblage aligns with broader patterns observed in Sudanese cuisine, where these crops have historically contributed essential nutrients. According to Nasreldein et al. (forthcoming), archaeobotanical research at Old Dongola revealed that sorghum, bread wheat, and hulled barley represent the most dominant cereal crops at the site, signifying their role as the primary carbohydrate source for the inhabitants of Old Dongola. The results show that sorghum composed the highest percentage of plant remains at the assemblage, representing 87% of ubiquity, followed by hulled barley, which represents 72% ubiquity, while bread wheat represents 34% ubiquity.

The presence of summer and winter crops forming the most consumed crops at Old Dongola aligns with the close proximity of the site to the Letti Basin, which provided fertile floodplain conditions ideal for surplus cultivation (Godlewski, 2013: 17–18), along with the narrow strips and the islands of the Nile. Additionally, the adoption of *saqiya* irrigation techniques (a cattle-powered waterwheel) further facilitated agricultural productivity and allowed for year-round cultivation in the region, enhancing the cultivation of drought-resistant crops like sorghum, as well as Mediterranean staples such as wheat and barley (Adams, 1977; AI-Batal, 1994: 47; Clapham, 2019: 90; Fuller, 2015: 41; Fuller & Lucas, 2020: 943; Obłuski, 2014a: 155; Trigger, 1970: 354). Consequently, the combination of environmental conditions and irrigation practices likely influenced the dominance of these cereals in the assemblage.

The integration of zooarchaeological evidence further enriches our understanding of dietary practices at Old Dongola. According to Osypińska and Godlewski (2020), the consumption of meat, particularly beef, mutton, goat, and camel, alongside wild game (gazelle and antelope) and Nile fish, contributed to the overall macronutrient composition of the Funj period diet at Old Dongola. The inclusion of animal products and the crops discussed in this paper likely shifted the proportions of macronutrients consumed by the Dongolese population between the fifteenth and sixteenth centuries toward higher proportions of carbohydrates. Osypińska and Godlewski (2020) indicated some variations in dietary habits at Old Dongola, such as the decline in pig consumption and the continued use of bird meat and reptiles, indicating dietary diversity influenced by cultural and environmental factors. The decline of pig consumption can be attributed to the Islamic rule forbidding pork. The data from this study show that legumes were a valuable part of protein intake. Nonetheless, this study does not have enough data to claim legumes replaced meat consumption.

Furthermore, the Old Dongola excavations revealed crop storage traces, represented by large storage jars (*gossiba*) and pits (*matmura*) for grains. The same house to which the discussed contexts belongs provided the only, so far, example of a storage pit (*matmura*) (1.5 m by 1.7 m and over 1.5 m deep) in Courtyard U73 (see Fig. 3) (Deptuła & Maślak, 2021: 61), but unfortunately, there was no evidence of stored crops inside the pit. The structure of the Funj houses at Old Dongola suggests an individual family-based economy, indicating that every household had its own storage space for food items (Obłuski & Dzierzbicka, 2021: 1, 2022: 242; Wyżgoł & Deptuła, 2023: 18). Based on the available evidence, it appears that the Funj houses at Old Dongola had a storage space behind the living room varying in size and capacity (Obłuski et al., 2022: 268; Obłuski & Dzierzbicka, 2021: 1, 2022: 242; Wyżgoł & Deptuła, 2023: 18). Dirar (1993: 389) classified this type of storage space as the most concealed storage area in the house, probably used to store valuable belongings, which is referred to in Sudanese Arabic as *gatee*. Furthermore, the design of the Funj houses included other storage space within the kitchens (Wyżgoł & Deptuła, 2023: 18), which was probably for food items and crops for daily base subsistence. The example we provide in this paper is likely one of the small storage spaces within the kitchen for daily consumption.

It is difficult to estimate the number of house dwellers at Old Dongola or to provide any estimations about the population that lived in the city. In this regard, further studies, such as that attempted by Wyżgoł (2022: 112–113), to estimate the number of people that could have been fed with the grain storage in some houses at Old Dongola during the Funj period, would provide valuable insights into population dynamics. Based on contemporary data regarding cereal grain consumption in Sudan during the second half of the twentieth century (Abdelrahman, 1998), Wyżgoł (2022: 112–113) argued that the estimated grain consumption per person at Old Dongola during the Funj period may have been similar and was approximately 149 kg per year, comprising mainly sorghum (106 kg), millet (28.6 kg), and wheat (14.4 kg). Further, Wyżgoł (2022: 112–113) indicated that the analysis of the abundance and volume of storage vessels that may have fitted in an oblong bench intended for holding storage vessels, compared to the annual needs of residents, indicates significant variation among houses, from large and small scale storage benches to no traces of storage at all at some houses. Wyżgoł (2022: 112–113) argued that if we consider that these storage vessels were meant to satisfy the needs of between5 and 17 adult individuals, this suggests that these resources were generally sufficient for the sustenance of a large family, assuming a family size similar to contemporary standards, which typically consist of around 6 people. Therefore, despite variations in storage capacity among households, the available resources were likely adequate for meeting the dietary needs of the inhabitants of Old Dongola during the Kingdom of Dongola period (Wyżgoł, 2022: 112–113).

Our findings support Wyżgoł’s (2022) conclusion regarding the adequacy of storage vessels for meeting the dietary needs of family-based subsistence at Old Dongola. We observed variations in storage capacity but found that the families likely used small storage elements (small vessels and basketry) to sustain their daily nutritional intake. Together, all these elements allow us to begin reconstructing an overview of the agricultural practices and subsistence of the Makurian and Funj periods at Old Dongola by investigating the stored crops and their implications for culinary practices.

## Conclusion

The presence of small-scale household storage at Old Dongola reflects one aspect of a broader economic system that likely included larger storage practices. Our study focuses specifically on the short-term storage practices that would have supported household-level subsistence strategies. Based on the size of the storage vessels and containers, it is evident that the inhabitants of Old Dongola adopted household-based, small-scale storage practices for cereals and crops. The presence of stored crops inside the houses suggests a household-based economy, where crop processing and storage were likely managed individually at the household level. This evidence indicates that pantry storage for daily consumption coexisted with storage intended for longer-term use, possibly for sowing or as surplus for extended periods. The variability in crop types and their distribution points to a flexible strategy that balanced immediate nutritional needs with future planting requirements. These findings suggest that short-term, small-scale household storage practices functioned as part of a complex and adaptive economic system rather than replacing larger storage solutions or communal storage methods.

Our findings reveal a blend of Mediterranean and African elements, reflecting the diverse cultural influences assimilated at Old Dongola. The presence of wheat and barley, despite the absence of ovens, suggests that these grains may have been adapted for preparation in alternative dishes similar to modern-day *gurassa*, *fateer*, or *madida*, alongside their possible use in porridges or boiled preparations. This adaptation illustrates how wheat and barley might have been integrated into established Sudanese culinary traditions despite technological changes in bread-making.

The study emphasizes the central role of sorghum as a staple in the Sudanese diet, both historically and in the present. Sorghum and its by-products played a significant role in various food preparations and beverages, serving as a dietary pillar across centuries. In contrast, crops such as wheat, barley, and legumes have received comparatively less attention in archaeological and ethnographic research. The discovery of charred legume remains at Old Dongola is particularly significant, as legumes are often poorly preserved unless specific conditions like charring are met. This finding highlights the likely importance of legumes in the ancient Sudanese diet, reinforcing their nutritional and cultural value.

The discovery of radish seeds in large quantities offers further insights into local agricultural and culinary practices. While these seeds may have been intended for oil extraction or as a stored food source, their presence also strongly suggests preservation for sowing in the next planting season. This evidence indicate the complexity of crop storage practices in the region, where strategies for both immediate consumption and future cultivation were employed.

In conclusion, this study provides valuable insights into subsistence and culinary practices at Old Dongola during the Funj period. The evidence suggests that local communities adopted a diverse dietary strategy, blending daily nutritional needs with the preservation of crops for future agricultural cycles. This flexible approach reflects the community’s ability to adapt to environmental, economic, and cultural changes while maintaining established culinary traditions.

## Data Availability

All data supporting the results and interpretations in this paper are presented within the main text, in the form of tables and figures. Archaeobotanical materials were excavated, exported to Germany, and analyzed at the Archaeobotany Laboratory of the University of Tübingen, under the permissions granted by the National Corporation for Antiquities and Museums of Sudan (NCAM). The raw data are currently housed at the Archaeobotany Laboratory, University of Tübingen, and are available upon reasonable request. Access to the raw data may require consultation with the repository and archiving department of the Polish Centre of Mediterranean Archaeology, University of Warsaw, as part of the UMMA project framework.
